# Anesthesia in Patients With Long COVID or Post-infectious Respiratory Sequelae Undergoing Emergency Surgery: Clinical Challenges and Perioperative Strategies

**DOI:** 10.7759/cureus.104067

**Published:** 2026-02-22

**Authors:** Sabrina Montoya, Diego Alvarez Ramirez, Ronald Chavarría, Elia L Zamora, Christian Andrés Soto Cordero

**Affiliations:** 1 Emergency Services, Hospital Monseñor Sanabria Martínez, Puntarenas, CRI; 2 Emergency Department, Hospital Monseñor Sanabria, Puntarenas, CRI; 3 Emergency Medicine, Hospital Monseñor Sanabria, Puntarenas, CRI

**Keywords:** anesthesia, emergency surgery, long covid, perioperative management, respiratory sequelae

## Abstract

The COVID-19 pandemic has left lasting health consequences that extend beyond the acute infection phase, with long COVID emerging as a complex multisystem condition that poses significant challenges in the perioperative setting. Patients with post-infectious respiratory or cardiovascular sequelae present an increased anesthetic risk due to persistent inflammation, pulmonary fibrosis, reduced lung compliance, and myocardial dysfunction. These alterations predispose to hypoxemia, arrhythmias, and hemodynamic instability during surgery, making preoperative assessment and individualized anesthetic planning essential. Comprehensive evaluation, including functional tests, cardiac and pulmonary imaging, and laboratory analysis, allows early identification of residual organ dysfunction that can compromise perioperative safety.

Anesthetic management must be adapted to the patient’s physiological condition, emphasizing lung-protective ventilation, cautious fluid therapy, and close hemodynamic monitoring. Regional anesthesia is preferred when feasible to minimize airway manipulation and reduce respiratory complications, while total intravenous anesthesia represents a safer option when general anesthesia is required. Postoperative care focuses on extended respiratory monitoring, multimodal analgesia to limit opioid use, and the implementation of pulmonary physiotherapy and antithrombotic prophylaxis to prevent complications. Psychological support is also recommended to address post-COVID anxiety and fatigue, contributing to holistic recovery.

Although clinical guidelines provide useful recommendations, current evidence remains limited and heterogeneous. Further research is required to clarify the pathophysiological mechanisms of long COVID, evaluate anesthetic drug interactions, and develop validated risk stratification tools. Establishing standardized, evidence-based perioperative protocols is essential to improve outcomes and ensure patient safety in individuals with long COVID undergoing emergency surgery.

## Introduction and background

The COVID-19 pandemic has had a profound impact on global health, with persistent post-infectious sequelae emerging as a major clinical concern. Long COVID, also referred to as post-acute sequelae of COVID-19 (PASC), is defined by the persistence of symptoms beyond three months after the initial infection. Clinical manifestations are heterogeneous and frequently include respiratory symptoms, such as dyspnea, chronic cough, and chest discomfort, as well as extrapulmonary involvement affecting the neurological and cardiovascular systems. Given its considerable global prevalence, long COVID represents an increasingly relevant condition in perioperative medicine, particularly in patients requiring anesthesia and surgical intervention [1,2].

Persistent respiratory impairment is among the most commonly reported sequelae. Abnormalities in diffusing capacity for carbon monoxide and radiological evidence of interstitial changes have been described in a subset of patients following hospitalization for COVID-19. Disordered breathing patterns and reduced exercise tolerance are also prevalent and may persist for months after the acute phase. In some cases, progressive or irreversible pulmonary alterations, including fibrotic patterns, necessitate systematic follow-up and periodic pulmonary function assessment to facilitate early detection and intervention [1,2].

The burden of long COVID among surgical candidates is clinically significant. Ongoing symptoms have been reported in a notable proportion of previously infected individuals, contributing to challenges in preoperative evaluation. Persistent abnormalities on pulmonary function testing and echocardiography have been observed and may correlate with reduced functional capacity and heightened perioperative risk. The heterogeneity of presentation complicates standardized assessment and underscores the need for structured preoperative evaluation protocols and multidisciplinary collaboration involving anesthesiologists, pulmonologists, cardiologists, and surgeons [3].

Beyond respiratory sequelae, the multisystemic nature of PASC necessitates a comprehensive perioperative approach. Residual myocardial inflammation, autonomic dysfunction, and impaired pulmonary reserve may influence anesthetic planning and intraoperative management. Lung-protective ventilation strategies, cautious fluid administration, and appropriate postoperative respiratory support are essential components of risk mitigation in this population [4]. A systems-based framework for perioperative assessment enables anticipation of intraoperative instability and postoperative complications, thereby enhancing patient safety and optimizing outcomes [5].

The objective of this study is to analyze anesthetic considerations, perioperative risks, and evidence-informed management strategies applicable to patients with long COVID or post-infectious respiratory sequelae who require emergency surgical intervention.

## Review

Methodology

This manuscript was designed as a narrative review aimed at providing a structured and clinically oriented synthesis of current evidence regarding anesthetic management in patients with long COVID or post-infectious respiratory sequelae requiring emergency surgery. The objective was not to conduct a systematic review or meta-analysis, but rather to integrate emerging literature into a practical perioperative framework.

A literature search was conducted in PubMed, Scopus, and Web of Science between January 2020 and March 2025. These databases were selected due to their broad indexing of peer-reviewed literature in anesthesiology, perioperative medicine, pulmonology, and critical care. The search strategy combined controlled vocabulary and free-text terms, including “long COVID” OR “post-acute sequelae of COVID-19” OR “PASC” AND “anesthesia” OR “perioperative management” OR “emergency surgery” OR “respiratory sequelae” OR “pulmonary dysfunction.” Boolean operators (AND/OR) were applied to refine relevance. Searches were limited to publications in English or Spanish.

Inclusion criteria comprised peer-reviewed original studies, clinical guidelines, systematic reviews, and relevant consensus documents published between 2020 and 2025 that addressed the pathophysiological mechanisms of long COVID, anesthetic considerations, perioperative risk stratification, respiratory impairment, cardiovascular involvement, or postoperative outcomes. Exclusion criteria included non-peer-reviewed material, editorials without clinical data, duplicate publications, and studies not directly related to perioperative implications of long COVID.

Titles and abstracts were screened for thematic relevance, followed by a full-text review when appropriate. Given the narrative nature of this review, no formal Preferred Reporting Items for Systematic Reviews and Meta-Analyses (PRISMA) flow diagram, quantitative pooling, or structured risk-of-bias assessment was performed. Instead, studies were selected based on clinical relevance, methodological clarity, and contribution to perioperative decision-making. A total of 34 documents were included and synthesized using a qualitative thematic approach.

Data extraction focused on identifying recurrent perioperative risk factors, organ-system involvement, monitoring strategies, and anesthetic management principles. Findings were organized into pathophysiological domains and clinical practice themes to enhance coherence and applicability. Artificial intelligence tools were used exclusively for linguistic refinement, structural organization, and editorial consistency. AI assistance did not influence study selection, interpretation of findings, or clinical conclusions.

The narrative design and absence of formal quantitative synthesis represent methodological limitations, which are acknowledged. However, this approach was considered appropriate given the heterogeneity of available evidence and the evolving nature of long COVID research.

Pathophysiological considerations

**Figure 1:**
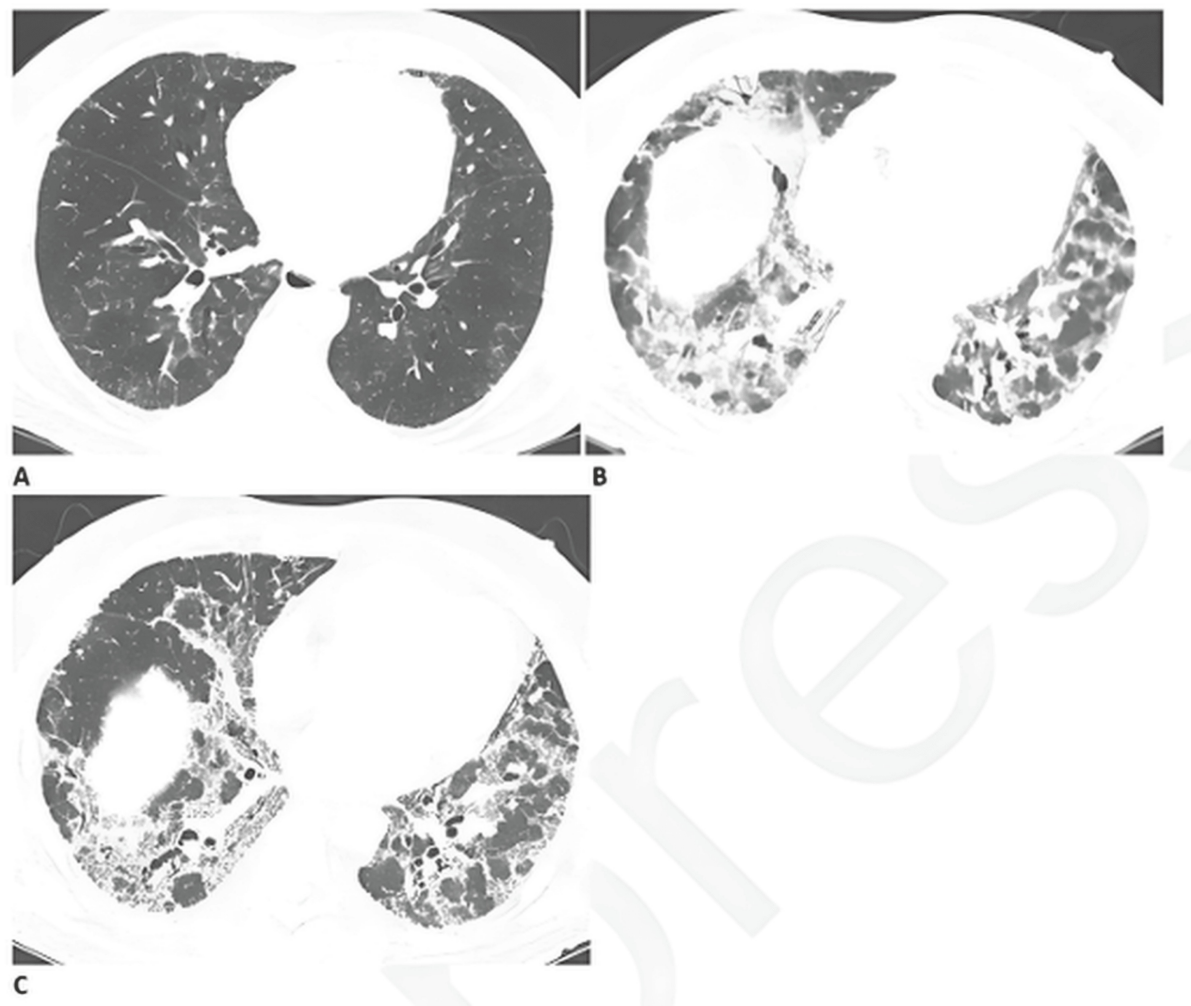
Progressive pulmonary fibrosis in a patient with post-infectious respiratory sequelae Progressive pulmonary fibrosis in a 67-year-old male with a prior history of relatively mild, stable fibrotic hypersensitivity pneumonitis. (A) Baseline computed tomography demonstrates mild ground-glass opacities and reticular changes. (B) Computed tomography angiography performed two months after infection reveals a marked increase in reticular abnormalities with mild traction bronchiectasis. (C) Follow-up computed tomography two months later shows further progression of traction bronchiectasis, consistent with advancing fibrosis. Figure reproduced from Solomon et al. (Licensed under Creative Commons Public Domain Mark 1.0.) [9].

From a respiratory standpoint, persistent inflammation and pulmonary fibrosis are frequent findings in post-COVID patients (Figure 1). These alterations lead to reduced lung compliance and an increased risk of ventilator-induced lung injury during surgery. To mitigate this risk, the implementation of lung-protective ventilation strategies, such as the use of low tidal volumes and individualized positive end-expiratory pressure (PEEP), is recommended [6,7]. Additionally, many patients exhibit impaired diffusion capacity and ventilation-perfusion mismatch, predisposing them to hypoxemia and hypercapnia. A comprehensive preoperative assessment of pulmonary function, along with close intraoperative monitoring of oxygenation and ventilation parameters, is essential to prevent complications [4,8]. Postoperatively, the risk of persistent hypoxemia and hypercapnia warrants the use of non-invasive respiratory support and continuous monitoring of arterial blood gases to ensure adequate recovery [4].

Cardiovascular considerations are equally important, as long COVID can cause myocardial inflammation and autonomic dysregulation, leading to arrhythmias and hemodynamic instability. These alterations require continuous cardiac monitoring during surgery and cautious administration of anesthetic agents that may exacerbate cardiac depression [4,10]. Furthermore, microthrombotic phenomena have been reported in patients with post-COVID sequelae, increasing the risk of perioperative thromboembolic events. Individualized assessment for anticoagulation therapy is therefore necessary to minimize these risks [11].

Beyond the cardiopulmonary system, long COVID may also affect neurological, hepatic, renal, and musculoskeletal function, each with implications for anesthetic care. Cognitive dysfunction, often described as “brain fog,” along with autonomic disturbances, can complicate anesthetic depth assessment and recovery. Tailored anesthetic plans that account for these neurological effects are essential [4]. Similarly, renal and hepatic impairment can alter drug metabolism and clearance, requiring preoperative evaluation and dose adjustment to prevent toxicity [10]. Lastly, muscular weakness and fatigue are common sequelae that may delay postoperative recovery, making early mobilization and structured rehabilitation strategies a key component of postoperative management [11].

Preoperative evaluation

A comprehensive clinical history provides critical information for understanding the patient’s current physiological condition and guiding perioperative decisions. It should include details about the severity and duration of the initial COVID-19 infection, vaccination status, and any ongoing symptoms, such as dyspnea, chest pain, or fatigue, which may suggest residual respiratory or cardiovascular compromise [3,4]. Evaluating functional capacity through objective measures, such as the six-minute walk test and continuous SpO₂ monitoring, offers valuable insights into the patient’s respiratory reserve and cardiovascular performance [12]. Equally important is the identification of residual symptoms, as persistent dyspnea, chest discomfort, or generalized fatigue might reflect ongoing organ dysfunctions that can significantly influence anesthetic and surgical outcomes [3,5].

Complementary laboratory and imaging assessments play a pivotal role in quantifying the physiological impact of long COVID (Figure 1). Pulmonary function tests, when feasible, are useful for detecting lingering respiratory impairments commonly observed in post-COVID patients [12]. Chest imaging, either by X-ray or computed tomography, is recommended to identify interstitial fibrosis, ground-glass opacities, or other residual pulmonary abnormalities. Given the frequent cardiovascular involvement in long COVID, echocardiography and electrocardiography are essential to detect myocardial inflammation, arrhythmias, or ventricular dysfunction [5,12]. Baseline oxygenation should also be evaluated through arterial blood gas analysis to uncover subtle hypoxemia or ventilatory inefficiency before anesthesia induction [13].

Risk stratification must integrate both traditional and post-COVID-specific considerations. The American Society of Anesthesiologists (ASA) physical status classification can serve as a foundation but should be adapted to account for the multisystemic impact of long COVID. The use of specialized perioperative scoring systems or individualized algorithms enhances the ability to predict complications and tailor management strategies for each patient [14,15].

Intraoperative management

The selection of anesthetic technique in patients with long COVID or post-infectious respiratory sequelae must be individualized, considering the degree of pulmonary compromise, the type of surgery, and the patient’s overall physiological condition. Regional anesthesia is generally preferred in patients with significant respiratory impairment, as it reduces the need for airway manipulation and consequently minimizes the risk of aerosol generation and respiratory complications. Additionally, regional techniques decrease opioid and muscle relaxant requirements, which can be advantageous in individuals with residual respiratory dysfunction [16]. However, general anesthesia may still be necessary depending on the surgical procedure, in which case the use of total intravenous anesthesia (TIVA) is recommended over inhalational methods, as it avoids airway irritation and potential immunosuppressive effects associated with volatile anesthetics [17]. Agents that cause prolonged respiratory depression should be avoided. TIVA using drugs such as remifentanil, propofol, or ketamine allows the maintenance of spontaneous ventilation and airway reflexes, thereby reducing the incidence of perioperative respiratory complications [18].

Intraoperative ventilation should follow lung-protective principles to prevent ventilator-induced injury and postoperative pulmonary complications. The use of low tidal volumes and adequate PEEP is essential to maintain alveolar stability and improve oxygenation [19]. Individualized PEEP settings, guided by oxygenation parameters and lung mechanics, have been shown to optimize gas exchange and reduce postoperative complications [20]. Careful control of oxygen and carbon dioxide levels is equally important to prevent hypoxemia and hypercapnia. Strategies such as preoxygenation and gentle induction help minimize the risk of desaturation during anesthesia induction, particularly in patients with limited pulmonary reserve [21].

Hemodynamic management represents another critical component of anesthetic care in this population. Continuous monitoring for hypotension and arrhythmias is vital due to the cardiovascular autonomic instability that can accompany long COVID. Fluid therapy should be individualized, maintaining adequate perfusion while avoiding fluid overload and the development of pulmonary edema, especially in patients with compromised respiratory function [22].

Infection control and operating room safety continue to be essential aspects of perioperative management, particularly in cases where active infection or residual viral shedding is suspected. Operating rooms should be equipped with high-efficiency air filtration systems, and negative-pressure environments are recommended when feasible. The use of personal protective equipment (PPE) by all staff members is mandatory to reduce the risk of viral transmission during aerosol-generating procedures [22,23]. Strict adherence to institutional infection control protocols, including correct donning and doffing of PPE, minimizing unnecessary personnel exposure, and maintaining environmental decontamination, is fundamental to ensuring the safety of both patients and healthcare workers [24].

Postoperative care

The immediate postoperative period in patients with long COVID or post-infectious respiratory sequelae requires close and extended monitoring due to the high risk of pulmonary and systemic complications. Extended observation in the Post-Anesthesia Care Unit (PACU) or Intensive Care Unit (ICU) is often necessary to ensure adequate respiratory function and hemodynamic stability. Continuous monitoring allows for the early identification of hypoxemia, bronchospasm, or respiratory fatigue. Noninvasive ventilation techniques, such as high-flow nasal cannula therapy or continuous positive airway pressure (CPAP), can be highly effective in managing postoperative hypoxemia and preventing respiratory failure, particularly in patients with limited pulmonary reserve [25,26]. While supplemental oxygen is frequently used to prevent desaturation, its administration must be carefully titrated to maintain optimal oxygenation without inducing hyperoxemia, which can exacerbate oxidative stress and impair recovery. Continuous peripheral oxygen saturation monitoring remains essential to balance these risks [27].

Effective pain management is another crucial component of postoperative care in this patient population. A multimodal analgesic approach is recommended to minimize opioid requirements and reduce the incidence of opioid-induced respiratory depression. Agents such as dexmedetomidine have demonstrated benefits in enhancing postoperative recovery and decreasing opioid consumption when incorporated into Enhanced Recovery After Surgery (ERAS) protocols [28]. The use of regional anesthesia techniques, including neuraxial or peripheral nerve blocks, can further decrease the need for general anesthetics and systemic opioids, thus reducing the risk of respiratory depression and limiting aerosol-generating interventions during the perioperative period [17,29].

Preventive strategies play a central role in improving postoperative outcomes and minimizing complications. Early mobilization and pulmonary physiotherapy should be encouraged to enhance ventilation, prevent atelectasis, and improve overall respiratory function [28]. Given the persistent hypercoagulable state observed in patients with prior SARS-CoV-2 infection, antithrombotic prophylaxis is strongly recommended to reduce the risk of venous thromboembolism and other thrombotic events [30]. In addition to physiological care, addressing psychological well-being is equally important. Many patients with long COVID experience anxiety, fatigue, and cognitive impairment, which can hinder recovery. Providing psychological support and counseling as part of comprehensive postoperative management can improve both emotional resilience and clinical outcomes [31].

Discussion

Current evidence and clinical guidelines emphasize the need for comprehensive perioperative management in patients with a history of COVID-19, particularly those presenting with long-term respiratory or cardiovascular sequelae. Individuals who experienced severe infection or required intensive care frequently exhibit residual organ dysfunction, most notably cardiac and pulmonary impairment. Accordingly, current recommendations advocate for detailed preoperative evaluations that include cardiac biomarkers, echocardiography, and spirometry to identify ongoing physiological compromise that could negatively influence surgical outcomes [12]. From an anesthetic perspective, regional techniques are preferred whenever feasible, as they minimize airway manipulation and reduce aerosol generation, thereby lowering the risk of viral transmission to healthcare personnel. This approach aligns with global efforts to enhance procedural safety during and after the pandemic [17,29]. Infection control remains a cornerstone of perioperative care, with guidelines stressing the importance of rigorous use of PPE, proper air filtration systems, and the establishment of dedicated COVID-19 operating rooms for emergency procedures [22].

Despite these advancements, significant limitations persist in the current body of evidence. A major concern is the heterogeneity among national and international guidelines regarding perioperative management of COVID-19 patients. Many existing recommendations are derived from expert consensus rather than supported by high-quality clinical trials, resulting in inconsistencies in practice [32]. Furthermore, longitudinal data on surgical outcomes in post-COVID patients remain limited. The available studies often include small sample sizes, heterogeneous populations, and short follow-up periods, which restrict the ability to generalize findings and develop standardized protocols [12,33].

There are several critical gaps in knowledge that warrant further research. The pathophysiology of long COVID remains incompletely understood, and a deeper understanding of the mechanisms underlying persistent organ dysfunction is essential to refine anesthetic management and improve surgical outcomes [33]. Developing validated risk stratification tools specifically designed for patients with long COVID could enhance perioperative planning and patient safety [12]. Additionally, more research is needed to investigate potential interactions between anesthetic agents and the residual systemic effects of COVID-19, particularly in patients with multiorgan involvement or chronic inflammation, to optimize pharmacological safety and efficacy [34].

## Conclusions

Long COVID poses a relevant and evolving challenge in perioperative medicine due to its potential multisystem involvement, particularly affecting respiratory and cardiovascular function. Thorough preoperative assessment and individualized anesthetic planning are essential to identify residual organ dysfunction and mitigate perioperative risk. A systems-based approach that incorporates lung-protective ventilation, careful hemodynamic management, and close postoperative monitoring may improve safety in this population.

Current recommendations remain largely based on heterogeneous and predominantly observational evidence. Given the narrative design of this review and the evolving nature of long COVID research, definitive perioperative protocols cannot yet be established. Further prospective studies are required to validate risk-stratification strategies and develop standardized, evidence-informed guidelines for anesthetic management in patients with long COVID.
